# Supplementary material for: Structural basis for the recognition and degradation of host TRIM proteins by Salmonella effector SopA
Source: Nat Commun. 2017 Jan 13;8:14004. doi: 10.1038/ncomms14004 (PMC5241803; doi:10.1038/ncomms14004)
Supplement: Supplementary Information — Supplementary Figures and Supplementary Table. [file ncomms14004-s1.pdf]

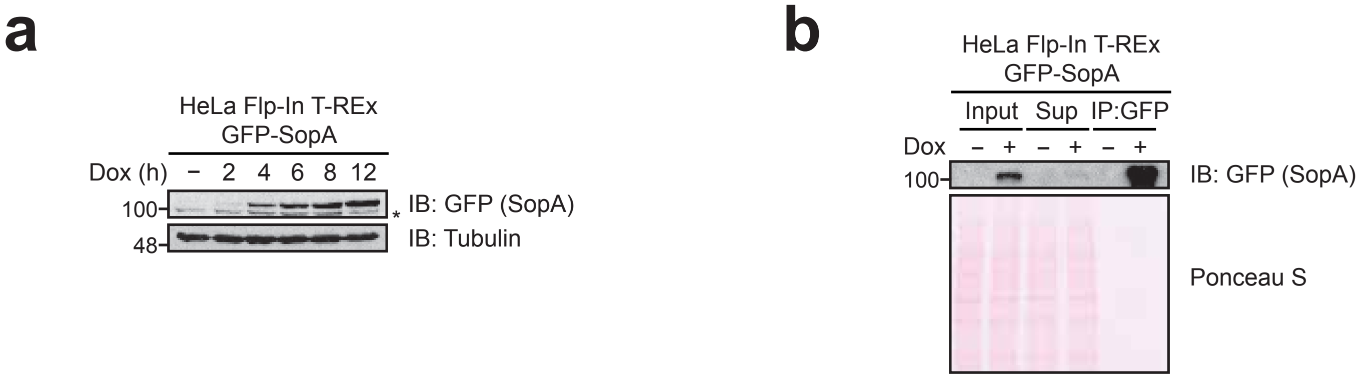

c

	# Peptides identified	Ratio (H/L) forward	Ratio (L/H) reverse	Intensity forward	Intensity reverse
SopA	8	18.018	14.116	530340000	127310000
TRIM56	23	15.301	15.119	36752000	187120000
TRIM65	7	16.830	10.857	19469000	19610000

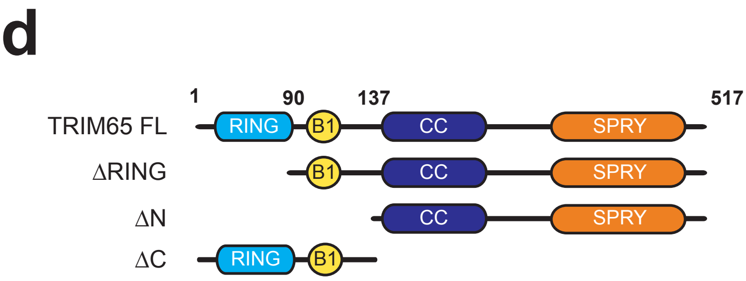

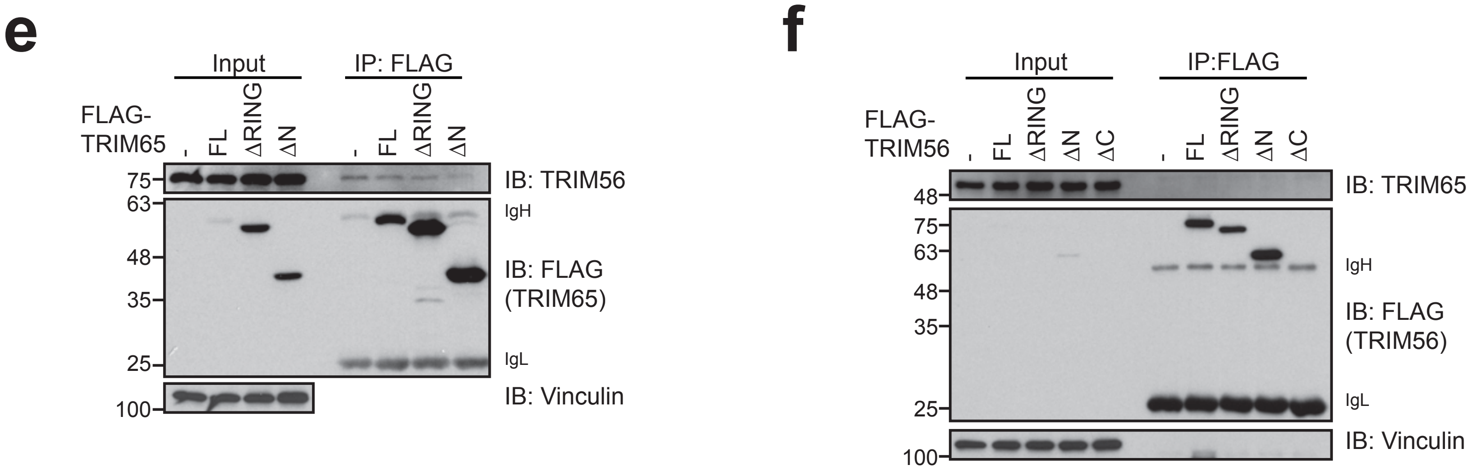

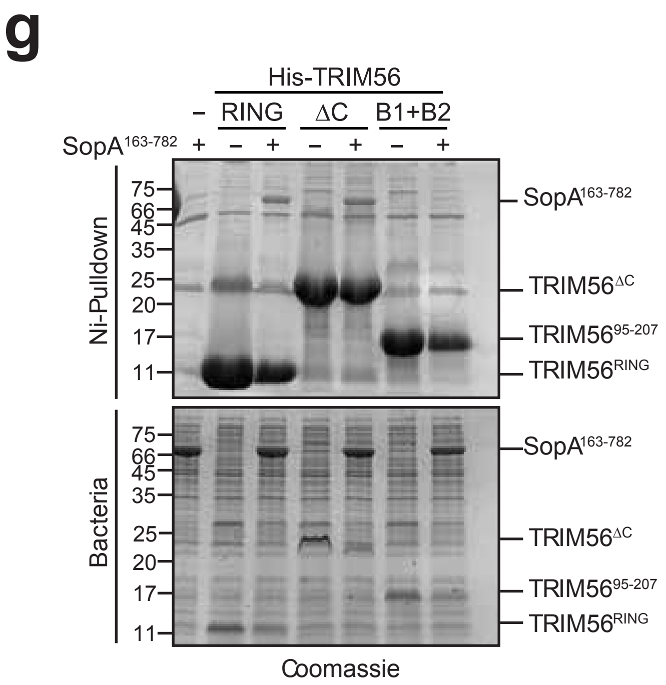

Supplementary Figure 1:
Interaction mode of SopA and TRIM56/65
(**a**) Stable cell line expressing GFP-SopA upon Doxycycline addition. Lysates of HeLa Flp-In T-REx GFP-SopA cells treated for different amounts of time with 1µg/ml Doxycycline to induce SopA expression were subjected to immunoblotting. (**b**) Purification of GFP-SopA from cells. HeLa Flp-In T-REx GFP-SopA were either left untreated or treated with 1µg/ml Doxycycline and GFP-SopA was immunoprecipitated using anti-GFP. IP supernatant and eluate were subjected to immunoblotting. (**c**) SopA SILAC interactome results. Number of identified SopA, TRIM56 and TRIM65 peptides, their ratios from swapped SILAC experiments (forward and reverse) and the corresponding sum of peptide intensities are shown. (**d**) Domain organization of TRIM65 and corresponding generated truncation constructs. (**e**,**f**) TRIM56 and 65 do not interact. Lysates from HEK293T cells expressing indicated FLAG-TRIM65 (**e**) or FLAG-TRIM56 (**f**) constructs were subjected to anti-FLAG IP, followed by SDS-PAGE and immunoblotting. (**g**) Untagged SopA (163-782) was subjected to nickel pull-down using indicated recombinant His-TRIM56 proteins.

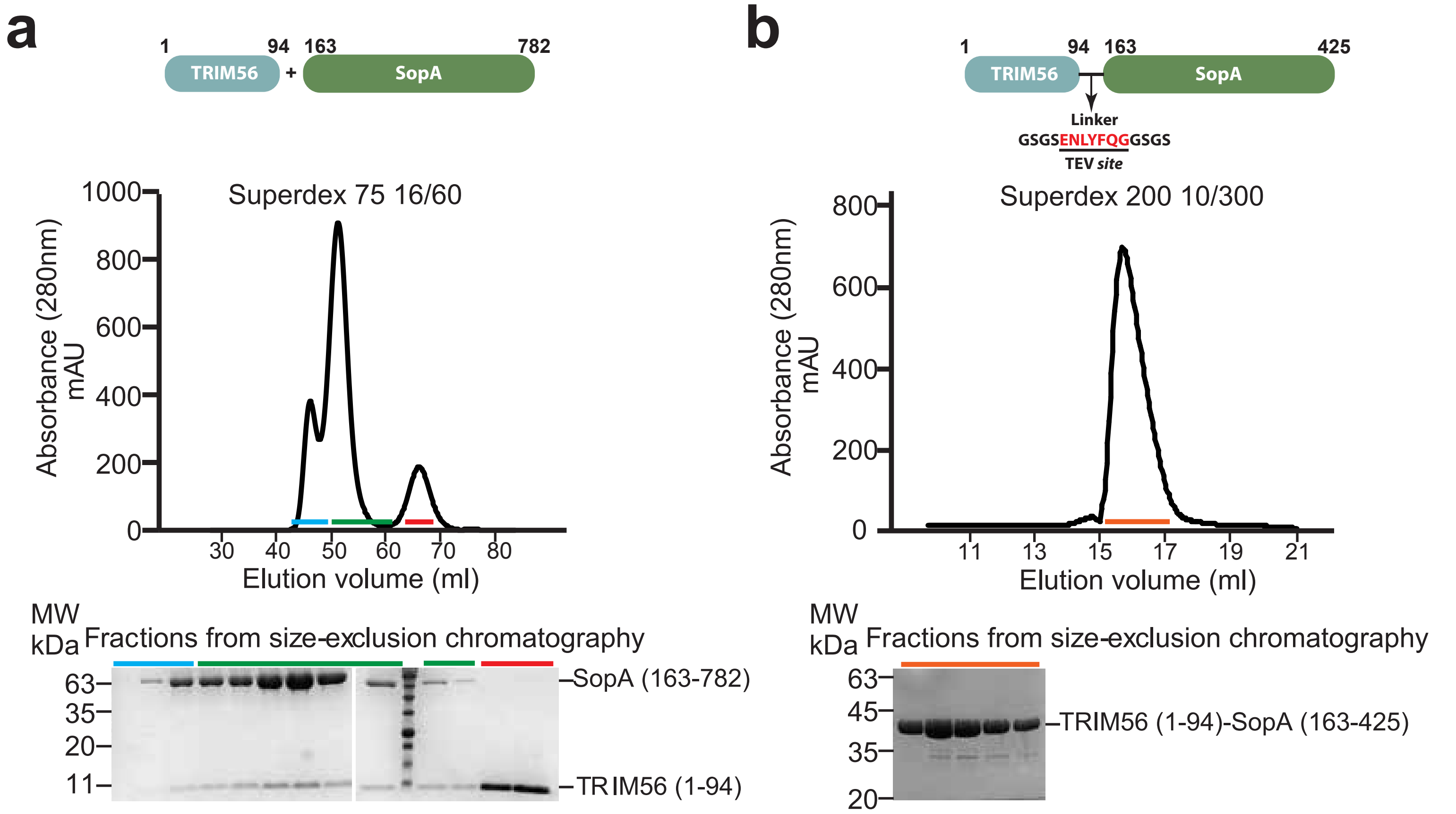

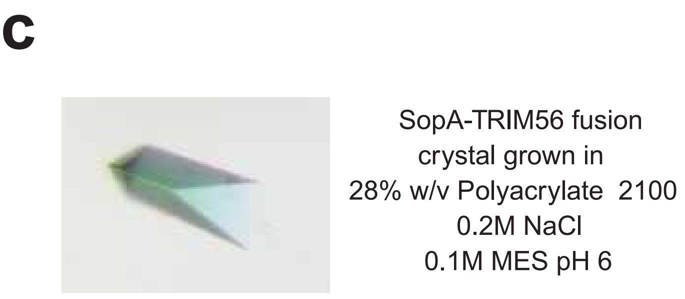

e

```
SopA 287 DTTLKNTLIRHKANLSG 303
NleL 301 EADLTGAIIIPGMVLSG 317
         :: *. ::*     ***

SopA 326 LGDIWLQMPLLWTENAVDGFLNHEHNNGKSIL 357
NleL 343 LSDIQEN---------ILSVLDNYTKSNKSIL 365
         *.**  :          : ..*:.  :. ****
```

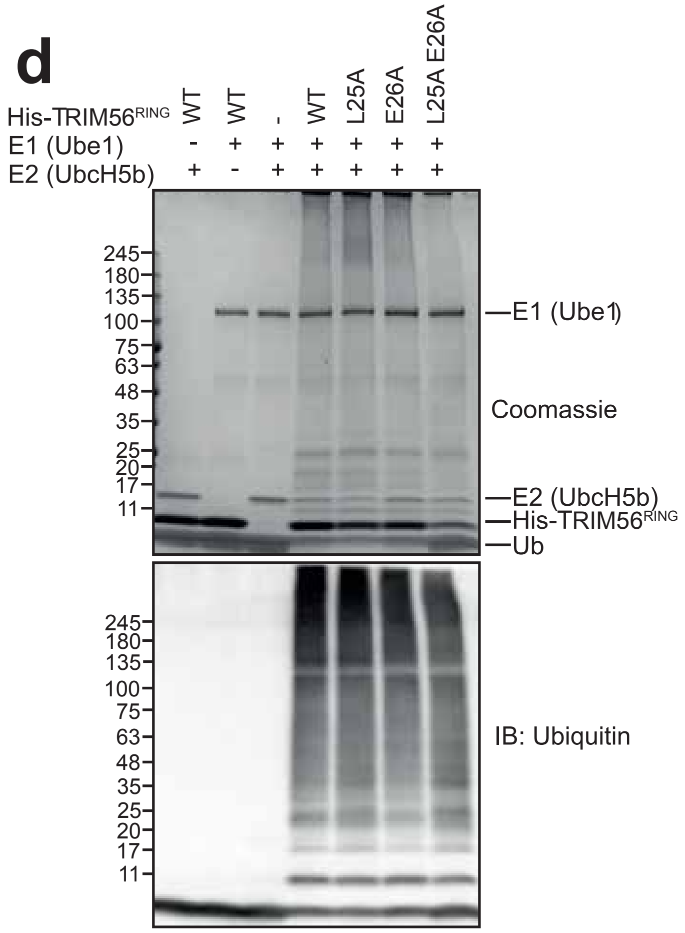

Supplementary Figure 2:
Purification and crystallization of SopA-TRIM56 complex
(**a**) Purified SopA (163-782) and TRIM56 (1-94) were mixed in equimolar concentrations and injected into Superdex-75 (16/60) size-exclusion column. Elution profile of the complex along with the corresponding coomassie stained SDS gel are shown. Unbound TRIM56 (1-94) elutes separately from SopA. (**b**) TRIM56 (1-94) and SopA (163-425) were fused using indicated linker sequence. The purified fusion protein was injected into Superdex-200 (10/300) size-exclusion column. Elution profile of the fusion protein along with the corresponding coomassie stained SDS gel are shown. (**c**) Crystallization condition for TRIM56-SopA fusion construct. (**d**) TRIM56 RING domain point mutants unable to bind SopA retain their catalytic activity. Purified WT or various mutant forms of HIS-tagged TRIM56 RING domain were mixed with purified E1, E2 (UbcH5b), and ubiquitin. (**e**) NleL lacks essential residues forming the SopA-TRIM56 interface. Sequence alignment of SopA and NleL residues spanning this region is shown.

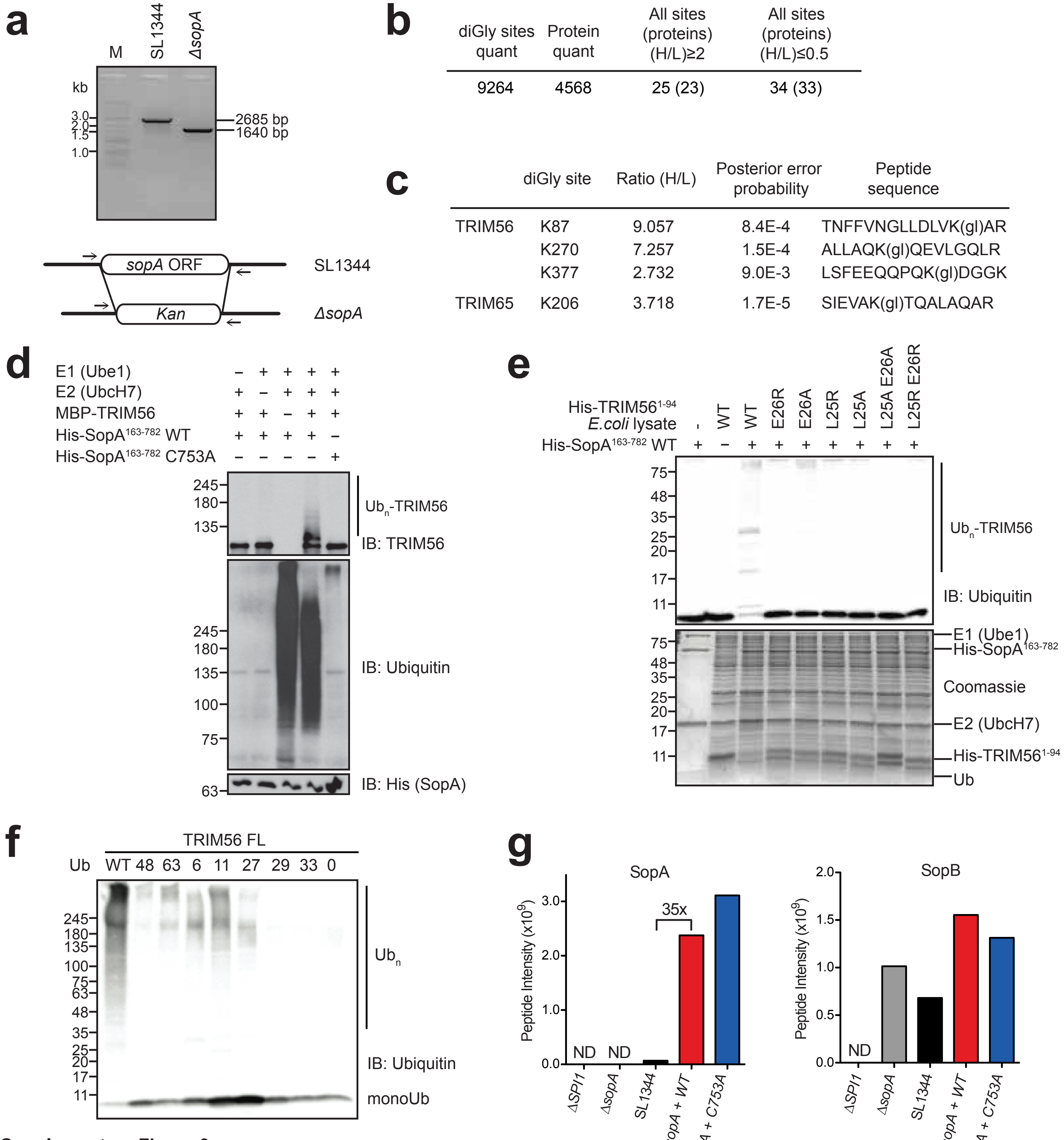

Supplementary Figure 3:
SopA-mediated ubiquitination and degradation of TRIM56/65

(**a**) Genotyping of SL1344 WT and *ΔsopA* strain using PCR amplification of bacterial genomic DNA with indicated primers (**b**,**c**) SL1344 WT versus *ΔsopA* SILAC diGly proteomics results. Total number of quantified ubiquitination sites and proteins from two replicates (**b**) and identified TRIM56 and TRIM65 diGly peptides with SILAC ratio and posterior error probability for identification are shown (**c**). (**d**) *In vitro* ubiquitination reactions of recombinant full-length MBP-TRIM56 and His-SopA (163-782) WT or catalytic-dead C753A were subjected to immunoblotting. UbcH7 served as E2 enzyme to exclude TRIM56 RING E3 activity. (**e**) *In vitro* ubiquitination of TRIM56 RING WT and various point mutants. Bacterial lysates expressing WT or various mutant forms of HIS-tagged TRIM56 RING domain were mixed with purified E1, E2 (UbcH7), SopA (163-782) and ubiquitin. SopA is able to ubiquitinate WT TRIM56 but not the binding interface mutants. (**f**) Ubiquitin chain linkage specificity of TRIM56. Purified full-length TRIM56 was incubated with E1, E2 (UbcH5b) and WT ubiquitin or various mutants of ubiquitin containing a single surface lysine residue. Samples blotted with ubiquitin antibody show that TRIM56 mainly synthesizes K63 and K11 chains in vitro. (**g**) SopA expression is elevated in *sopA*-reconstituted *sopA* deletion strains compared to endogenous levels in the SL1344 strain. Peptide intensities of secreted SopA, SopB and SopE effectors in HeLa cells infected with indicated *Salmonella* strains are shown.

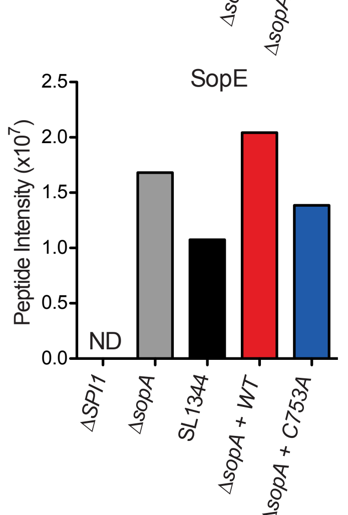

a

	# Peptides identified	Ratio (H/L) forward	Ratio (L/H) reverse	Intensity forward	Intensity reverse
TRIM28	8	1.193	1.578	5554300	2344700
TRIM32	24	0.690	0.948	968700	6325100
TRIM41	3	0.642	0.767	4077600	2282500

	# Peptides identified	Ratio (H/L) forward	Ratio (L/H) reverse	Intensity forward	Intensity reverse
TRIM26	45	1.329	0.964	62754000	71004000
TRIM2	12	1.304	1.147	3428100	1798600
TRIM32	11	1.167	0.886	15417000	8717500
TRIM25	14	1.124	1.048	7566100000	6731300000
TRIM14	1	1.101	0.695	5971300	4086400
TRIM3	6	1.100	1.030	247730000	185530000
TRIM27	13	0.975	0.571	2301300	3774100
TRIM28	2	0.921	0.941	2238200000	2917100000
TRIM21	33	0.868	0.901	18849000000	19601000000

b

	non-regulated diGly sites
TRIM11	141, 258
TRIM23	501
TRIM24	341
TRIM25	283, 310, 320, 335, 345, 416, 425, 439, 509
TRIM28	31, 261, 304, 319, 337, 340, 365, 366, 377, 400, 407, 434, 469, 695, 750, 779
TRIM32	175, 182
TRIM4	356
TRIM44	314
TRIM47	230, 361
TRIM59	286
TRIM72	149, 398

c

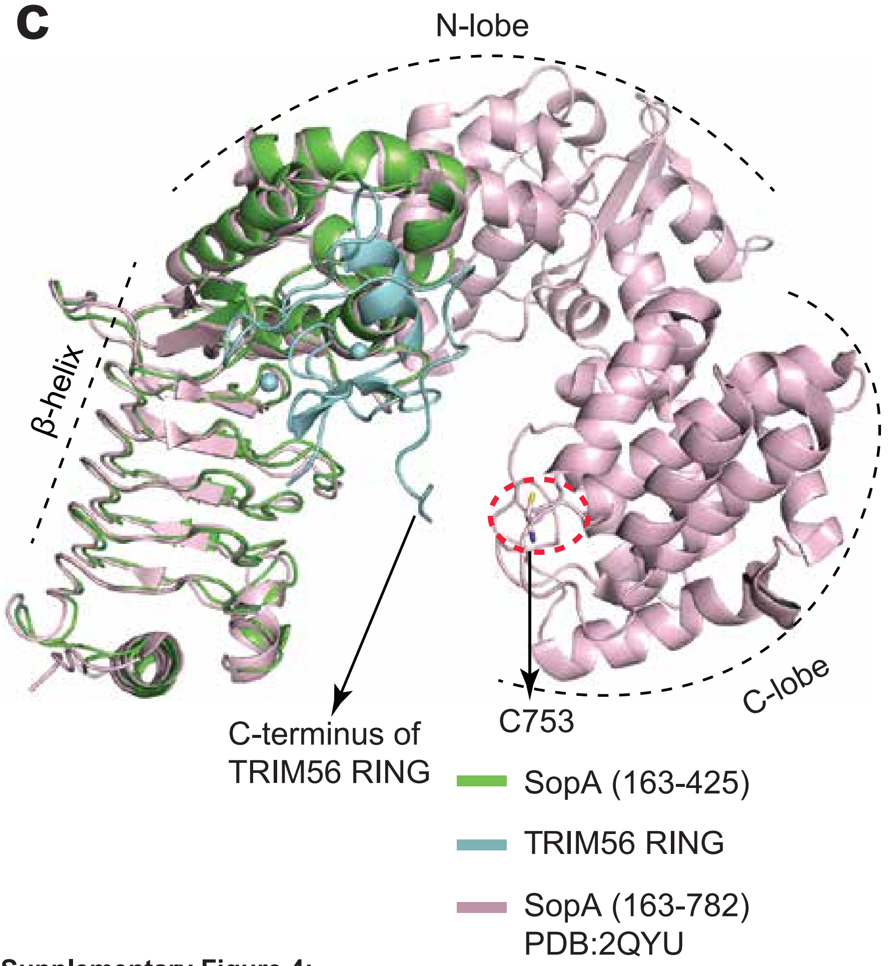

d

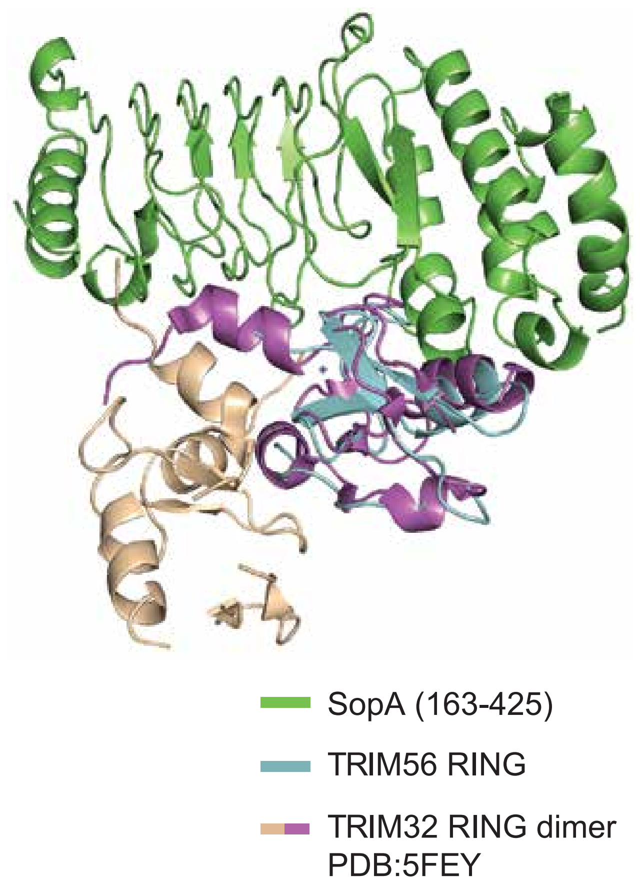

Supplementary Figure 4:
Proximity of SopA active site to TRIM56 acceptor lysine 87 and compatibility of SopA-binding with TRIM56 dimerization
(**a**) TRIM proteins, which are not interacting with SopA. Number of identified TRIM peptides, their ratios from swapped SILAC interactome experiments and the corresponding sum of peptide intensities are shown (top: data from GFP-SopA interactome Fig.1b; bottom: data from SopA-HA interactome upon infection Fig.1d). (**b**) Identified non-SopA-regulated TRIM diGly sites (data from SopA diGly proteomics Fig.6b). (**c**,**d**) Superimposition of SopA (163-425)-TRIM56 RING (1-94) complex structure with SopA (163-782) (**c**) and TRIM32 dimer structures (**d**). SopA catalytic cysteine C753 and TRIM56 ubiquitination site at Lys87 are highlighted.

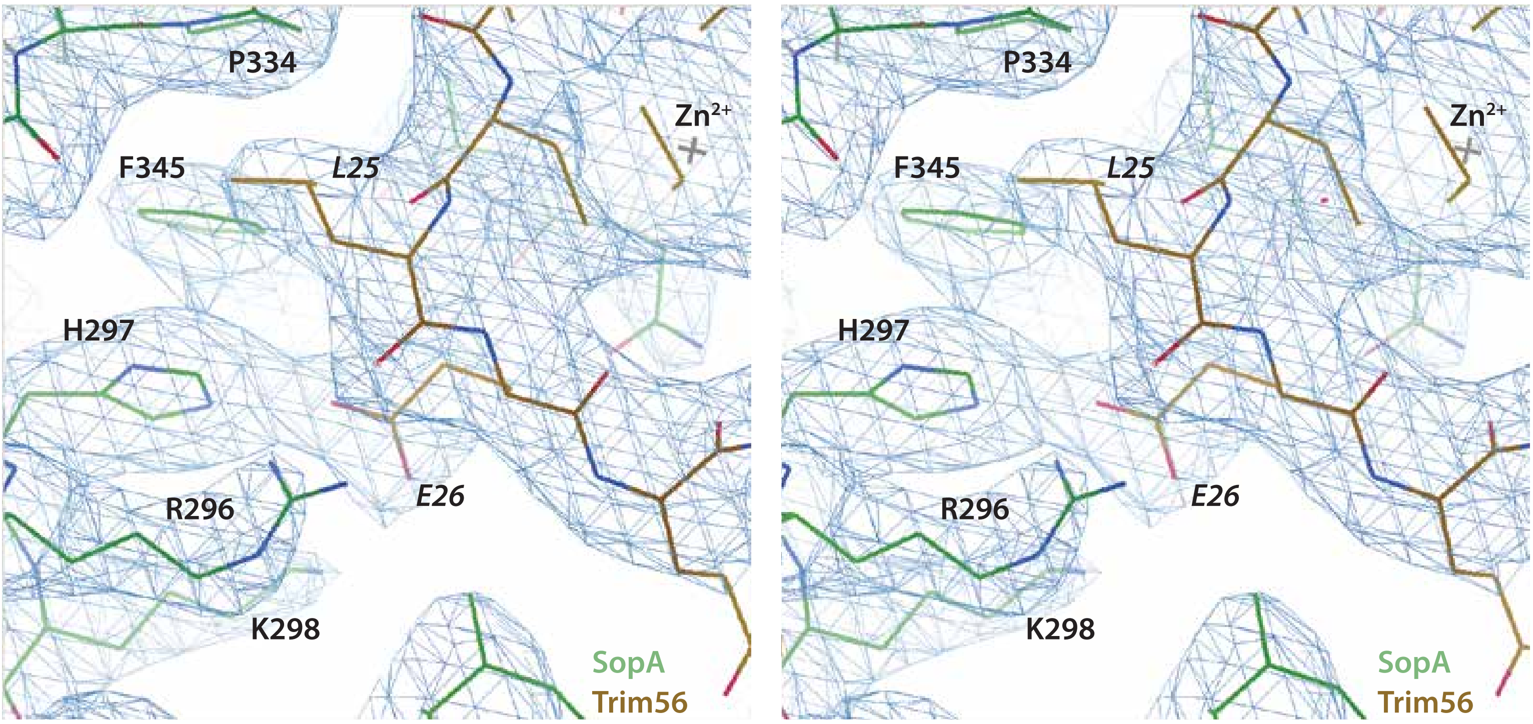

Supplementary Figure 5:
Side by Side stereo view of SopA-Trim56 crystal structure with 2Fo-Fc electron density (sigma level rendered to 1)
The figure was generated using Coot. Residues shown are at the interface of SopA and Trim56.

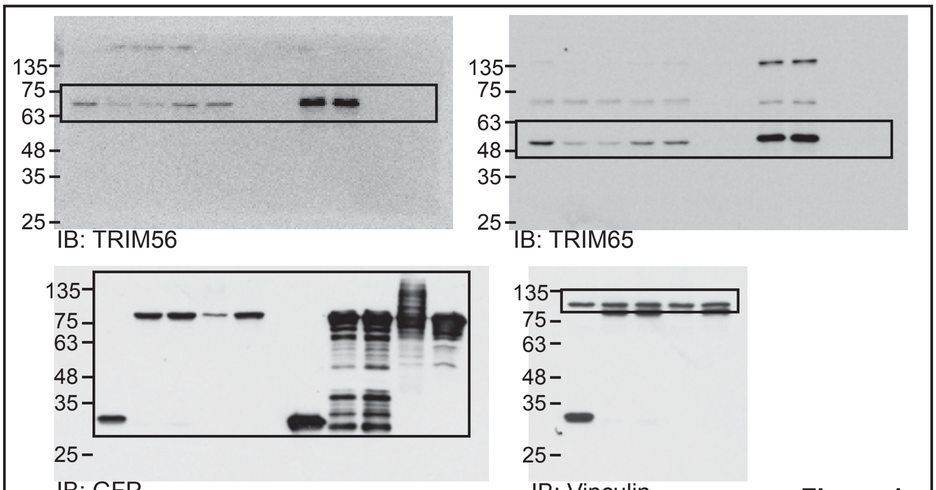

Figure 1c

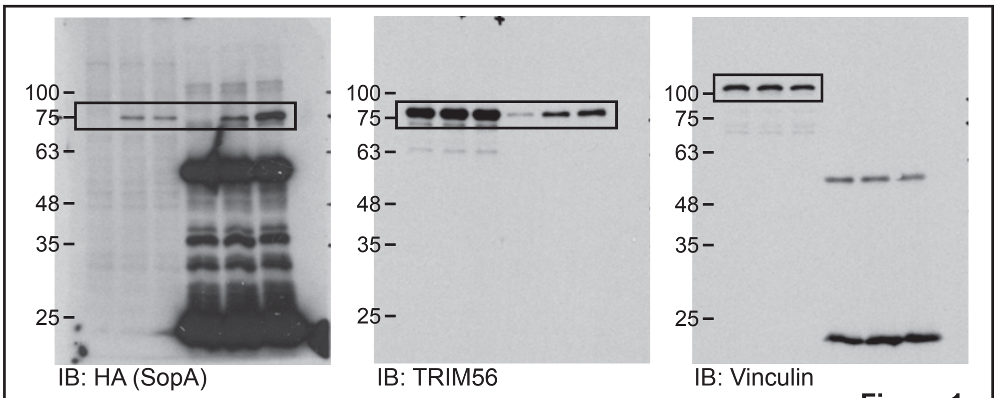

Figure 1e

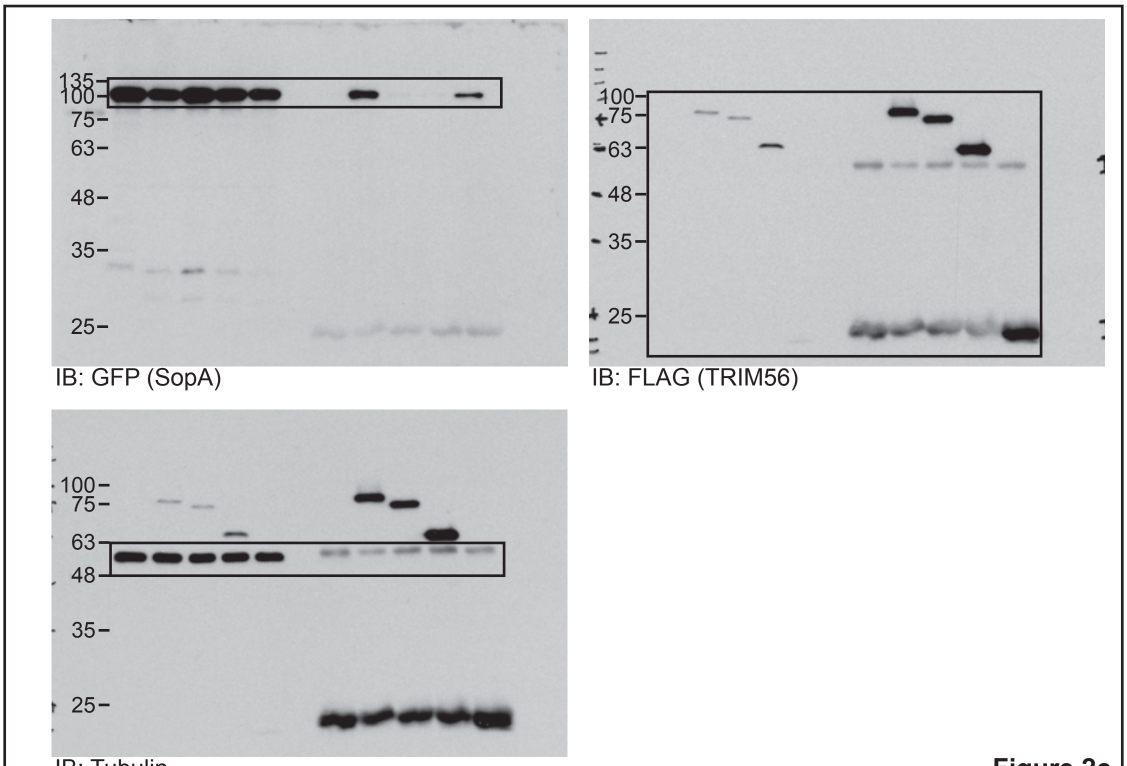

Figure 2c

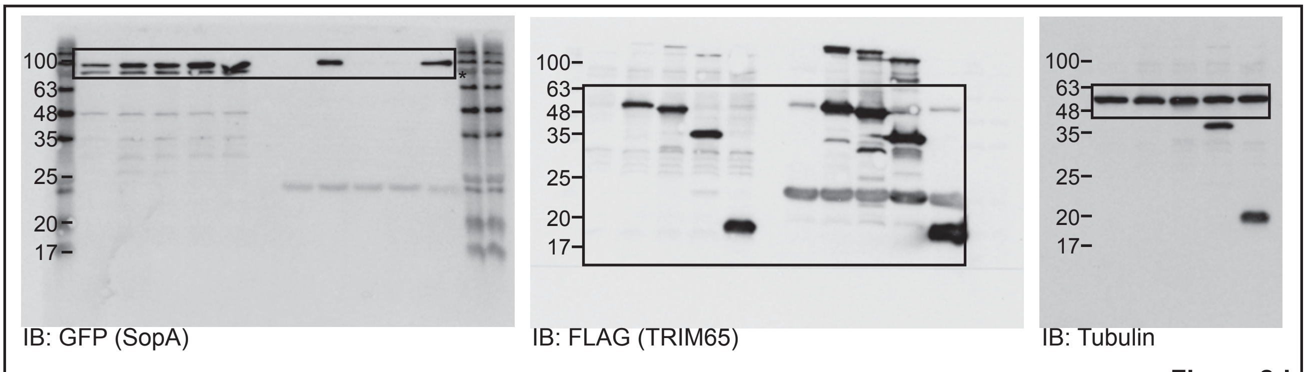

Figure 2d

Supplementary Figure 6: Uncropped Western blot for figures 1c, 1e, 2c and 2d

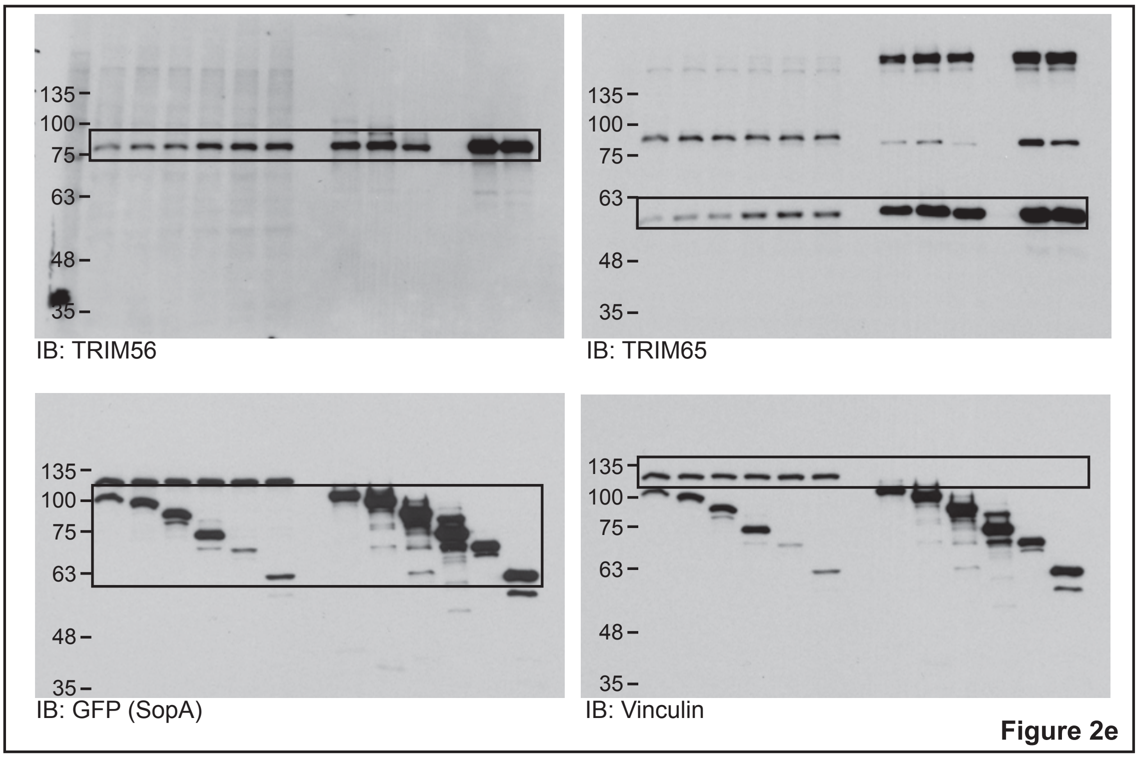

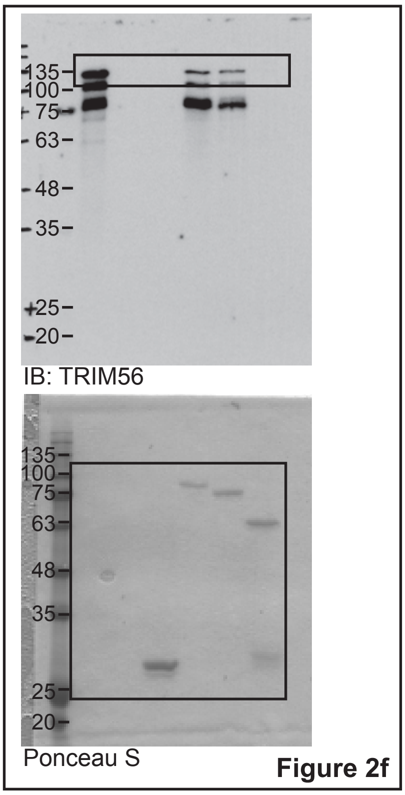

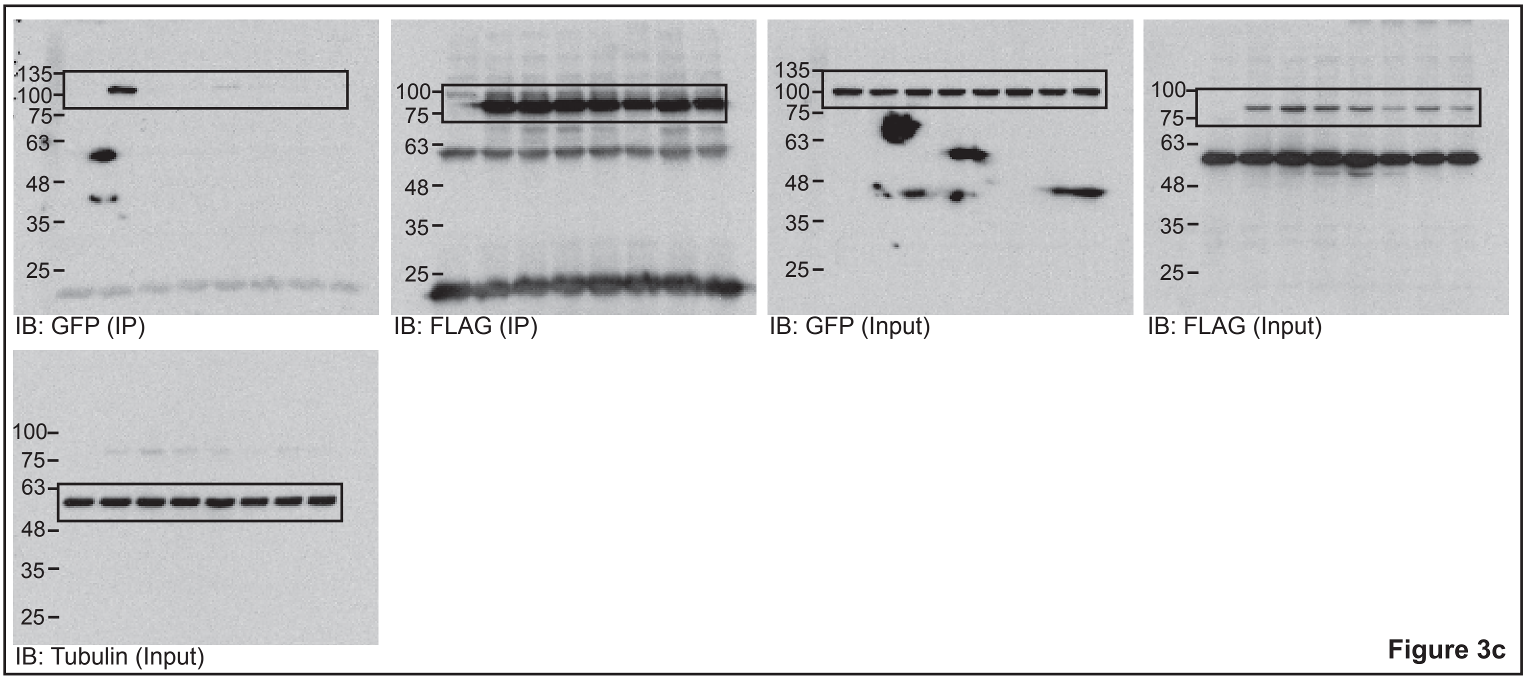

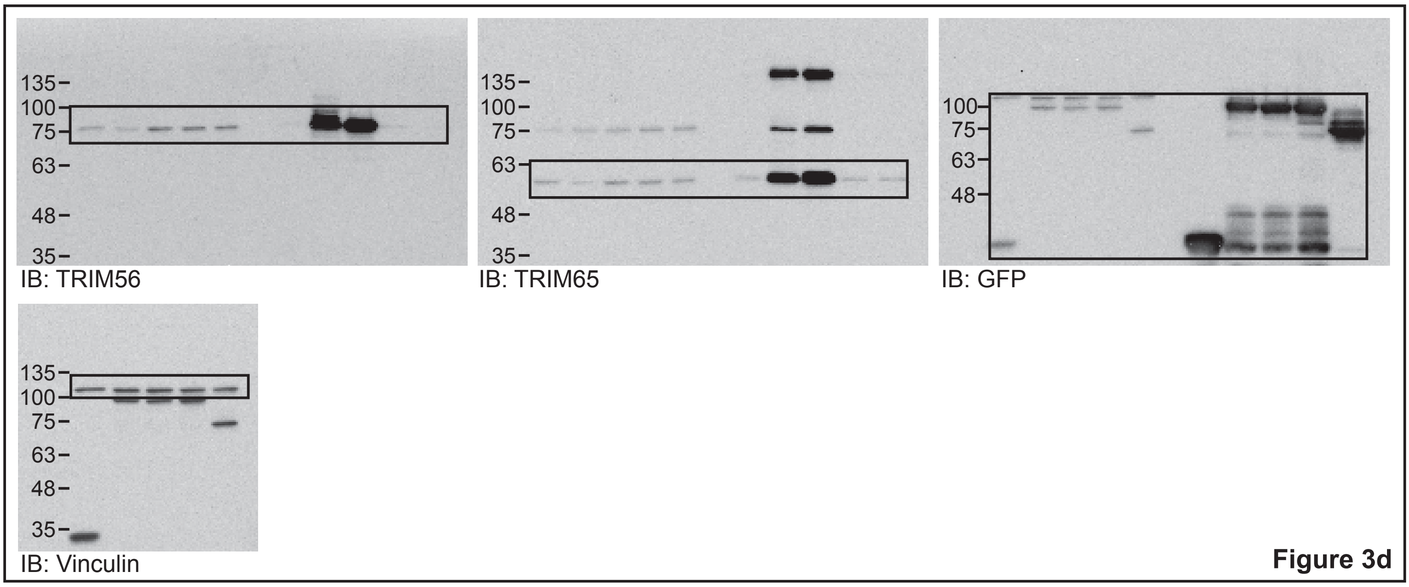

Supplementary Figure 7: Uncropped Western blot for figures 2e, 2f, 3c and 3d

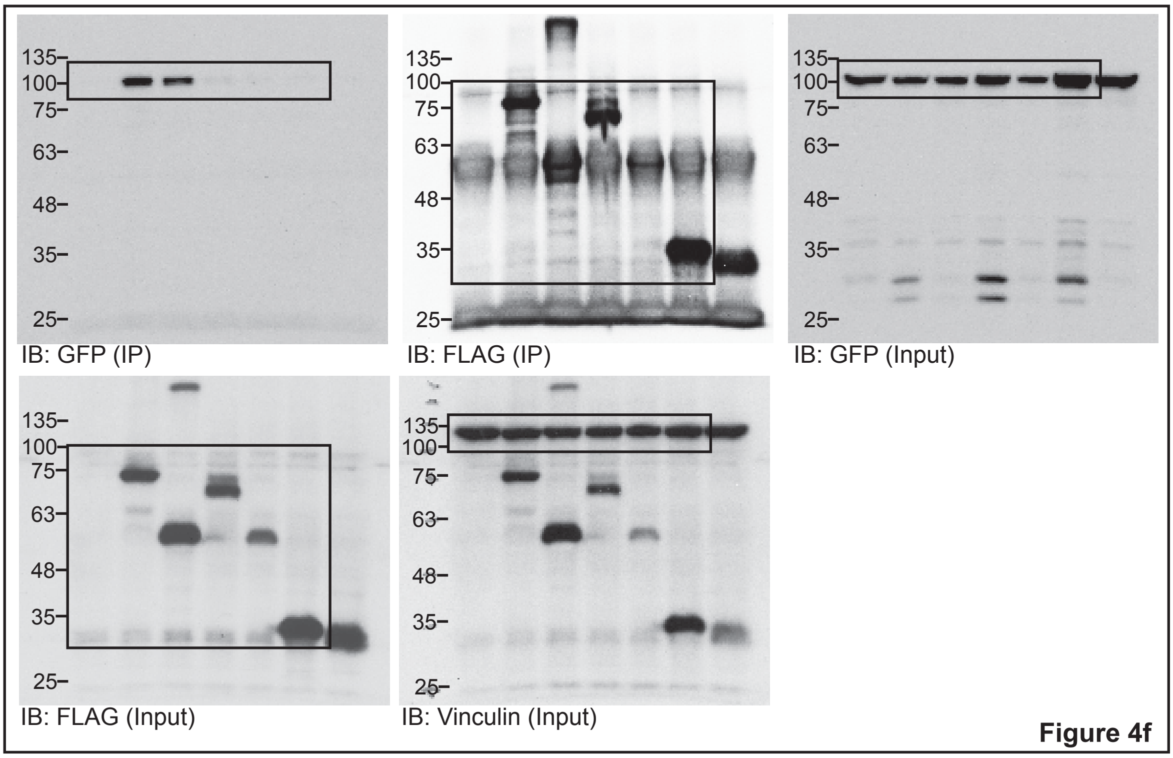

Figure 4f

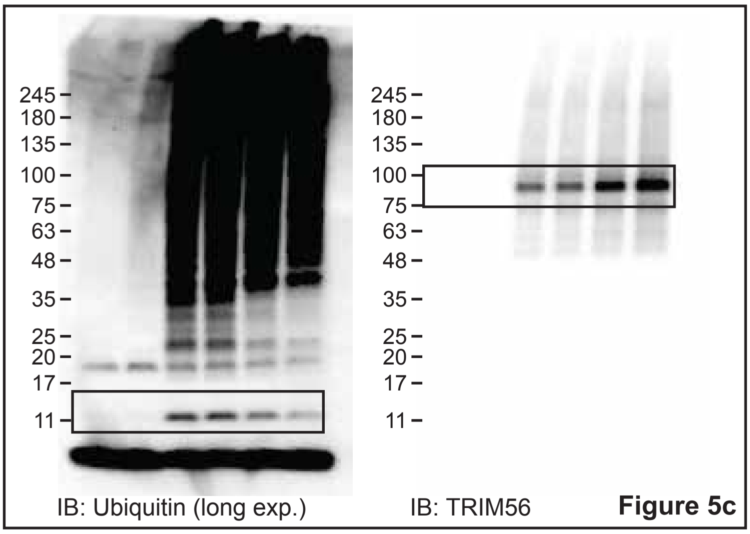

Figure 5c

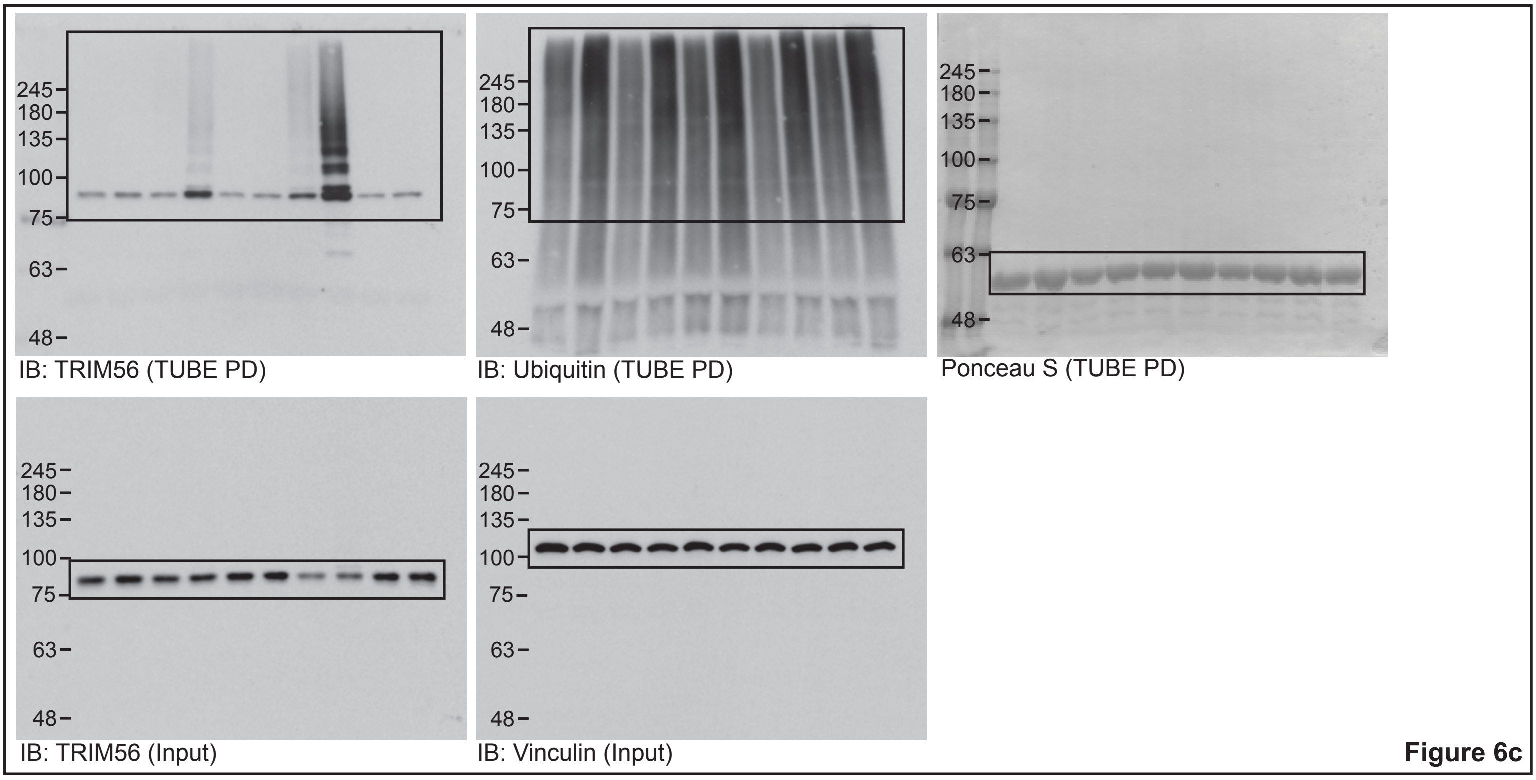

Figure 6c

Supplementary Figure 8: Uncropped Western blot for figures 4f, 5c and 6c

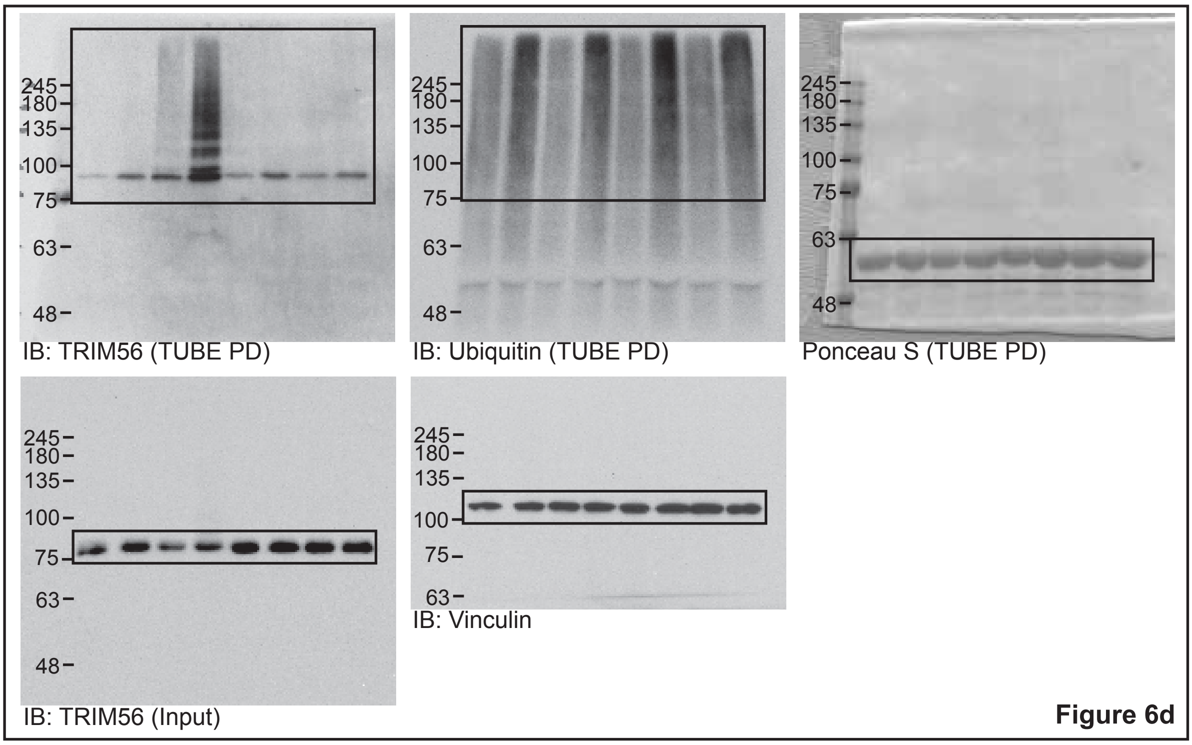

Figure 6d

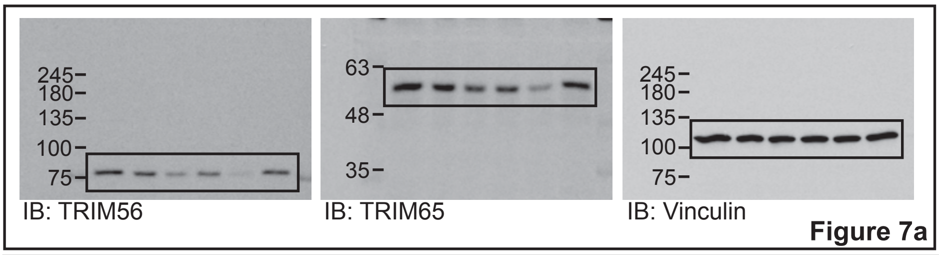

Figure 7a

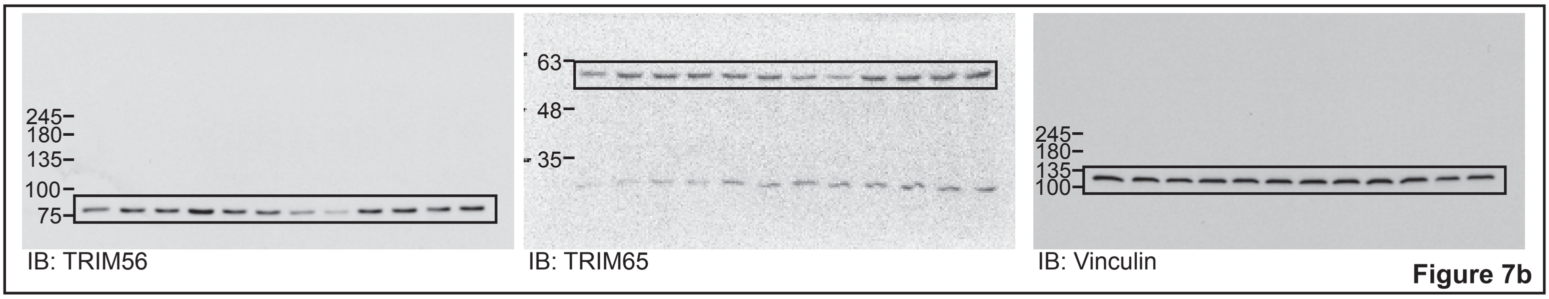

Figure 7b

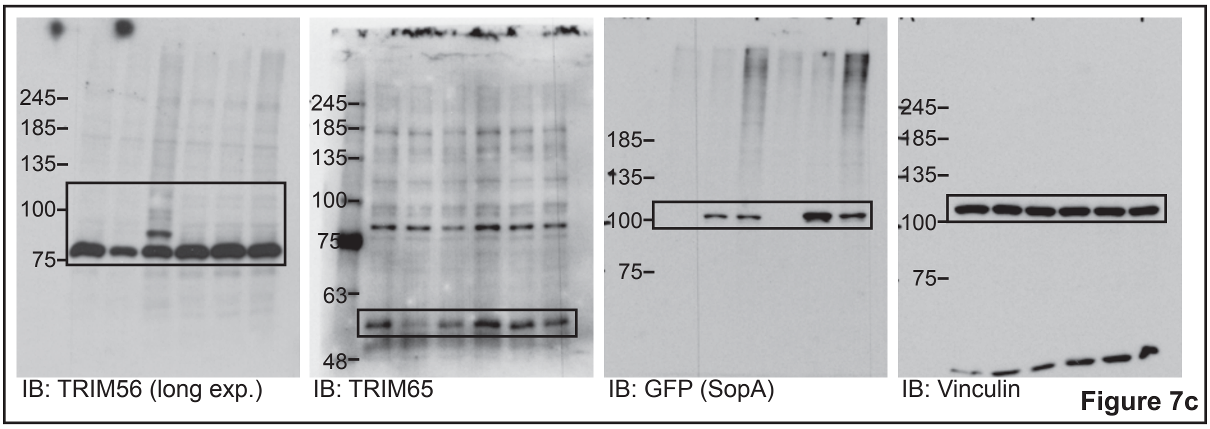

Figure 7c

Supplementary Figure 9: Uncropped Western blot for figures 6d, 7a, 7b and 7c

Supplementary Table 1: Plasmids and bacterial strains used in this study

Vector	Description
pcDNA5 FRT TO GFP-SopA	Full length N-terminally EGFP-tagged SopA under Tet-inducible CMV promoter
pcDNA5 FRT TO GFP-SopA C753A	Full length N-terminally EGFP-tagged SopA catalytic-dead Cys 753 to Ala mutant under Tet-inducible CMV promoter
pEGFP-C1 SopA	Full length N-terminally EGFP-tagged SopA
pEGFP-C1 SopA C753A	Full length N-terminally EGFP-tagged SopA catalytic-dead Cys 753 to Ala mutant
pEGFP-C1 SopA 56-782	N-terminally EGFP-tagged SopA lacking first 55 amino acids
pEGFP-C1 SopA 163-782	N-terminally EGFP-tagged SopA lacking first 162 amino acids
pEGFP-C1 SopA 307-782	N-terminally EGFP-tagged SopA lacking amino-terminus and beta helix domain
pEGFP-C1 SopA 56-470	N-terminally EGFP-tagged SopA encompassing amino acids 56-470 lacking catalytic HECT domain
pEGFP-C1 SopA 163-470	N-terminally EGFP-tagged SopA encompassing amino acids 163-470 lacking catalytic HECT domain
pEGFP-C1 SopA T338L	Full length N-terminally EGFP-tagged SopA Thr 338 Leu mutant deficient in Trim56/65 binding
pWSK29 SopA-HA	C-terminally HA-tagged SopA under its native SL1344 promoter
pWSK29 SopA C753A-HA	C-terminally HA-tagged SopA catalytic-dead Cys 753 to Ala mutant under its native SL1344 promoter
pWSK29 SopA	Untagged SopA under its native SL1344 promoter
pWSK29 SopA C753A	Untagged SopA catalytic-dead Cys 753 to Ala mutant under its native SL1344 promoter
pGEX-6P1 SopA 56-782	N-terminally GST-tagged SopA lacking first 55 amino acids
pGEX-6P1 SopA 163-782	N-terminally GST-tagged SopA lacking first 162 amino acids
pGEX-6P1 SopA 370-782	N-terminally GST-tagged SopA lacking amino-terminus and beta helix domain
pcDNA5 FRT TO FLAG-Trim56	Full length N-terminally FLAG-tagged human Trim56 under Tet-inducible CMV promoter
pcDNA5 FRT TO FLAG-Trim56 ΔRING	Full length N-terminally FLAG-tagged human Trim56 encompassing amino acids 64-755 lacking RING domain under Tet-inducible CMV promoter
pcDNA5 FRT TO FLAG-Trim56 ΔN	Full length N-terminally FLAG-tagged human Trim56 encompassing amino acids 208-755 lacking RING and B boxes under Tet-inducible CMV promoter
pcDNA5 FRT TO FLAG-Trim56 ΔC	Full length N-terminally FLAG-tagged human Trim56 encompassing amino acids 1-207 lacking coiled-coil and WD40 domain under Tet-inducible CMV promoter
pcDNA5 FRT TO FLAG-Trim56 L25A	Full length N-terminally FLAG-tagged human Trim56 Leu 25 to Ala mutant deficient in SopA binding under Tet-inducible CMV promoter
pcDNA5 FRT TO FLAG-Trim56 L25R	Full length N-terminally FLAG-tagged human Trim56 Leu 25 to Arg mutant deficient in SopA binding under Tet-inducible CMV promoter
pcDNA5 FRT TO FLAG-Trim56 E26A	Full length N-terminally FLAG-tagged human Trim56 Glu 26 to Ala mutant deficient in SopA binding under Tet-inducible CMV promoter
pcDNA5 FRT TO FLAG-Trim56 E26R	Full length N-terminally FLAG-tagged human Trim56 Glu 26 to Arg mutant deficient in SopA binding under Tet-inducible CMV promoter
pcDNA5 FRT TO FLAG-Trim56 L25A E26A	Full length N-terminally FLAG-tagged human Trim56 Leu 25 and Glu 26 to Ala mutant deficient in SopA binding under Tet-inducible CMV promoter
pcDNA5 FRT TO FLAG-Trim56 L25R E26R	Full length N-terminally FLAG-tagged human Trim56 Leu 25 and Glu 26 to Arg mutant deficient in SopA binding under Tet-inducible CMV promoter
pMAL-C2X Trim56	Full length N-terminally MBP-tagged human Trim56
pcDNA5 FRT TO FLAG-Trim65	Full length N-terminally FLAG-tagged human Trim65 under Tet-inducible CMV promoter
pcDNA5 FRT TO FLAG-Trim65 ΔRING	Full length N-terminally FLAG-tagged human Trim65 encompassing amino acids 55-517 lacking RING domain under Tet-inducible CMV promoter
pcDNA5 FRT TO FLAG-Trim65 ΔN	Full length N-terminally FLAG-tagged human Trim65 encompassing amino acids 139-517 lacking RING and B box under Tet-inducible CMV promoter
pcDNA5 FRT TO FLAG-Trim65 ΔC	Full length N-terminally FLAG-tagged human Trim65 encompassing amino acids 1-138 lacking coiled-coil and SPRY domain under Tet-inducible CMV promoter
pcDNA5 FRT TO FLAG TRIM32	Full length N-terminally FLAG-tagged human Trim32 under Tet-inducible CMV promoter
pET15b Trim56	Full length N-terminally HIS tagged human Trim56
pET21a Trim56 1-94	First 94 amino acids of Trim56 with C-terminal CPD-HIS tag
pET15bTrim56 1-94 E26A	First 94 amino acids of Trim56 with N-terminal HIS tag and a 3C cleavage site, mutant deficient in binding to SopA
pET15b Trim56 1-94 L25A	First 94 amino acids of Trim56 with N-terminal HIS tag and a 3C cleavage site, mutant deficient in binding to SopA
pET15b Trim56 1-94 E26R	First 94 amino acids of Trim56 with N-terminal HIS tag and a 3C cleavage site, mutant deficient in binding to SopA
pET15b Trim56 1-94 L25R	First 94 amino acids of Trim56 with N-terminal HIS tag and a 3C cleavage site, mutant deficient in binding to SopA
pET15b Trim56 1-94 E26R L25R	First 94 amino acids of Trim56 with N-terminal HIS tag and a 3C cleavage site, mutant deficient in binding to SopA
pET15b Trim56 1-94 E26A L25A	First 94 amino acids of Trim56 with N-terminal HIS tag and a 3C cleavage site, mutant deficient in binding to SopA
pET21a Trim56 1to94 fused to SopA 1to 425	Fusion contruct of SopA trim56 used for crystallization with C-terminal CPD-HIS tag
pET26b SopA 163-782	Untagged SopA used in co-expression studies with Trim56 constructs
pET15b Trim56 1-207	First 207 amino acids of Trim56 with N-terminal HIS tag and a 3C cleavage site
pET15b Trim56 95-207	95-207 amino acids of Trim56 with N-terminal HIS tag and a 3C cleavage site
pET28a UbcH7	Human UbcH7 with a C-terminal HIS tag
pET28a Ube1	Mouse Ube1 with a C-terminal HIS tag
pET21a SopA 163-782	SopA 163-782 with a C-terminal CPD-HIS tag
pET15b SopA 163-782	SopA 163-782 with an N-terminal HIS tag

Bacterial strains	Description and Source
SL1344 WT	wild-type *Salmonella enterica* serovar Typhimurium strain
ΔSPI1	SL1344 InvA deletion leading to ablation of SPI1 Type 3 secretion system; kind gift from Jorge Galan
ΔSopA	SL1344 SopA deletion using lambda red recombination with Kanamycin resistance; this study
ΔSopA+empty	SL1344 SopA deletion complemented with pWSK29 empty vector; this study
ΔSopA+SopA	SL1344 SopA deletion complemented with pWSK29 SopA; this study
ΔSopA+SopA C753A	SL1344 SopA deletion complemented with pWSK29 SopA catalytic dead Cys 753 Ala mutant; this study
ΔSopA+SopA T338L	SL1344 SopA deletion complemented with pWSK29 SopA Thr 338 Ala mutant; this study
ΔSopA+SopA-HA	SL1344 SopA deletion complemented with pWSK29 SopA-HA; this study
ΔSopA+SopA C753A-HA	SL1344 SopA deletion complemented with pWSK29 SopA-HA catalytic dead Cys 753 Ala mutant; this study
